# Editorial: Tissue Engineering for Drug Discovery and Personalized Medicine

**DOI:** 10.3389/fmedt.2021.663057

**Published:** 2021-02-26

**Authors:** Qasem Ramadan, Parveen Sharma

**Affiliations:** ^1^College of Science & General Studies, Alfaisal University, Riyadh, Saudi Arabia; ^2^Department of Cardiovascular and Metabolic Medicine, University of Liverpool, Liverpool, United Kingdom

**Keywords:** 3D bioprinting, tissue engineering, regenerative medicine, drug discovery, *in vitro*

During the past century, animal models have provided a wealth of knowledge and understanding of the mechanism of human disease and therapy and played a vital role in medicine and drug discovery. However, the use of these models in research and industry remains the focus of intense ethical debate and brought further into focus by government agencies and research bodies who have endorsed the implementation of the 3R's initiative aimed at the Replacement, Reduction, and Refinement of animals in all areas of research. Coupled to this, the lack of translational research often found in animal models that have led to several examples where toxicity and damage have not been found in animal models and efficacy either over or underestimated has led to costly and sometimes fatal drug failures during human clinical studies. The current trend in translational pharmaceutical research is focused on finding alternative methods that rely on complex human factors and conditions. Recent advances in cell biology, tissue engineering, and microsystem technologies, such as microfluidics and 3D bioprinting, have enabled devising innovative solutions by creating biomimetic physiological tissue structures and environments. The transdisciplinary integration of bioengineering, cell biology, and tissue engineering is paving the way toward moving from the traditional 2D single-cell models to larger multi-cellular structures, such as organoids and Micro-Physiological Systems (MPS) aka organs-on-a-chip (OOC), and engineering niches that able to control organ/organoid development as reliable *in vitro* models for drug screening in the pre-clinical stages.

The past two decades have seen a rapid growth of MPS technology, which aims to boost the development of drug discovery and personalized medicine. Multiple organ models are now being biologically integrated onto single chips which would expand the possibilities for assessing drug toxicity and efficacy within an *in vivo*-like environment. Furthermore, these developments would enable us to investigate how cells interact, differentiate, and respond to external stimuli in health and disease states, identifying possible drug targets.

This Research Topic is addressing this task by including the most relevant work in pharmaceutical applications of tissue engineering. There are two original research papers and four reviews which highlight the new advances in this field with an emphasis on the interface between the technological advancements and high impact applications.

Cox et al. discussed the use of spheroids, organoids, and perfusion-based systems to establish suitable liver models for investigating metabolism-dependent drug-induced liver injury (DILI), including their metabolic capabilities. These adverse drug reactions can often involve interactions between different cell types, which are also being modeled *in vitro* using co-culture systems. The authors indicated that *in vitro* liver models for investigating and detecting DILI-positive compounds require the use of metabolically competent cells that are able to recapitulate the function of the human liver *in vivo*. This enables the detection of mechanistically relevant toxicities that are translatable to the clinic and drug development, which in turn can prevent the unnecessary attrition of drugs and testing on animals during the drug development pipeline. Ramadan and Zourob reviewed the recent progress of the 3D bioprinting technology and discussed how the integration of 3D printing and tissue engineering, is paving the way toward devising many innovating solutions for key healthcare challenges including pharmaceutical, regenerative medicine, and food industries. Salminen et al. reviewed the cellular and molecular events involved in leukocyte and cancer cell extravasation focusing on *in vitro* studies of transendothelial migration (TEM) and highlighted the potential of applying these platforms for pharmaceutical compound screening. In addition, the authors discuss the promise of hepatocyte transplantation to restore liver function.

The blood-brain barrier (BBB) is one of the most important histological barriers with unique properties that control the communication and molecular trafficking between the Central Nervous System (CNS) and the periphery. Williams-Medina et al. gave a historical perspective on *in vitro* models of the BBB and the recent 3D models and the potential of uses these models to study human brain diseases and their treatments with an emphasis on NeuroAIDS, COVID-19, Multiple Sclerosis, and Alzheimer's disease as examples of *in vitro* model application. Finally, Shin et al. proposed a methodology that enables reproducible attachment of intestinal organoid epithelium in a PDMS-microfluidic device by polyethyleneimine-based surface functionalization followed by the glutaraldehyde cross-linking to activate the PDMS surface. The experimental results show this protocol provides uniform ECM deposition that leads to a robust attachment of the dissociated intestinal organoid epithelium to the PDMS surface.

This collection of papers provides excellent contributions with the state-of-the-art and critical reviews on the recent advances in the field of microphysiological systems in drug discovery and its impact applications. We are grateful to the authors who contributed to this Research Topic and pleased to witness the great progress in this arena.

## Author Contributions

All authors listed have made a substantial, direct and intellectual contribution to the work, and approved it for publication.

## Conflict of Interest

The authors declare that the research was conducted in the absence of any commercial or financial relationships that could be construed as a potential conflict of interest.

